# Physical exercise behavior characteristics and influencing factors for participation in police officers

**DOI:** 10.3389/fpubh.2024.1507066

**Published:** 2025-01-03

**Authors:** Yuliang Feng, Chao Chen, Yang Liu, Sen Li

**Affiliations:** ^1^Police Training Department, Shanghai Police College, Shanghai, China; ^2^School of Physical Education, Dalian University, Dalian, China; ^3^School of Physical Education, Nanchang Normal University, Nanchang, Jiangxi, China; ^4^School of Physical Education and Health, Shanghai Lixin University of Accounting and Finance, Shanghai, China

**Keywords:** physical fitness, exercise, motivation, influencing factors, law enforcement

## Abstract

**Objective:**

The purpose of this study was to analyze police officers’ exercise participation behavior and influencing factors in order to better promote physical exercise participation and improve the health status of the police force.

**Methods:**

Police officers (*n* = 3,682) were recruited from 16 district public security substations in a city in eastern China to investigate their physical exercise participation and influencing factors, and logistic regression analysis was used to investigate three aspects: exercise behavior, health cognition, and influencing factors.

**Findings:**

The physical exercise behavior of police officers showed long duration of time, but low frequency and intensity. The exercise programs were diverse but mainly involved running. The exercise motivation was mainly to keep fit and for stress relief. Logistic regression analysis indicated that source of police entry (OR = 1.467, 95% CI: 1.338–1.609), exercise conditions in the workplace (OR = 1.069, 95% CI: 1.051–1.088), exercise conditions near home (OR = 1.123, 95% CI: 1.047–1.205), high volume physical labor during work resulting in little time for exercise (OR = 1.372, 95% CI: 1.038–1.813), lack of organization (OR = 1.415, 95% CI: 1.164–1.720), economic condition constraints (OR = 1.439, 95% CI: 1.114–1.858), and weather restrictions (OR = 2.929, 95% CI: 2.418–3.548) were the main factors influencing police physical exercise behavior.

**Conclusion:**

Various influencing factors and the nature of actual police work presented barriers to police officers’ participation in physical exercise. Interventions to increase physical exercise participation would promote the physical health of police officers.

## 1 Introduction

Police officers are expected to perform a physically demanding task as part of their potentially hazardous career, whilst upholding their duties to protect the public and prevent crimes (1). Based on this occupational trait, police officers must be physically capable of performing all the demands of the profession and maximizing the safety of the community and those involved (2, 3). Therefore, the physical ability of police officers is closely related to their job performance, both high levels of physical ability and physical exercise are necessary in order to perform well when on duty, partly because of the frequent need to chase and detain suspects (4). Physical exercise behavior refers to people’s awareness of the use of leisure, sports, means and methods to seek physical and psychological health or accomplish some other purpose of physical activity under the interaction between the internal and external environment (5). Studies have shown that physical exercise, as well as leisure-time exercise, plays a major role in physical performance and health (6), and health cognition has an impact on exercise behavior (7). Police work is hard, complicated, and stressful. In police officers’ limited spare time, if they can participate in some sports and cultural activities, they can achieve both physical and mental adjustment (8, 9). Police officers’ engagement in physical exercise is important for several reasons: to deal with work-related situations, to take care of their health, and to strengthen psychological wellbeing (10).

Previous studies have revealed physical activity patterns in policing work time (11), motivational regulation of police college students (12), physical activity levels in police officers and cadets (13), and if self-efficacy for exercise is predictive of leisure-time physical activity (14). Previous studies about physical exercise have mainly focused on teenagers or the ordinary adult population, and the findings concerned motivation, exercise programs, and influencing factors (15, 16). However, research on police exercise behavior has mainly focused on frequency and quantity, the relationship between police physical fitness and the frequency of leisure-time physical exercise (17), or physical activity, fitness, and the body composition of police officers (18). Cognition determines behavior, while behavior lead to outcomes and is influenced by some internal and external factors. Current research is lacking on the characteristics of physical exercise behavior of police officers, the degree of health cognition, and the influencing factors. These issues need to be solved urgently to eliminate barriers to exercise and promote the physical exercise behavior of civilian police, so as to enhance physical fitness and improve police work ability.

Therefore, this study investigated and analyzed the characteristics and influencing factors of physical exercise behavior of police officers to provide insights on the physical health of police officers and the optimal development of police careers.

## 2 Materials and methods

### 2.1 Study design and setting

This study recruited a total of 3,682 police officers to participate in the investigation, including 3,236 males and 446 females. This study was approved in February 2023 by the Ethics Committee of Shanghai University of Sport (No. 102772023RT186). The questionnaire for this study was developed by referring to the relevant literature (19–21). The questionnaire was tested for reliability and consistency, and the overall Cronbach alpha value was 0.951, indicating a high reliability of the questionnaire. The survey questionnaire consisted of four parts and 30 questions. The first part was the basic information of the subjects, including gender, age, workplace, education, and source of police entry. The second part was exercise behavior, which referred to the Physical Activity Rating Scale PARS-3 (22). The setting included the exercise duration, exercise intensity, frequency of exercise and main physical exercise items. The third part was health cognition, which referred to the Physical Fitness Belief Scale (23). The setting included exercise motivation, self-assessment of current physical condition, and understanding of the importance of physical activity. The last part was the influencing factors, including exercise conditions, exercise place, exercise time, and constraints.

The dependent variable was “whether to participate in physical exercise regularly” (1 = yes, 0 = no), and the independent variables were gender, age, education, source of joining the police, exercise conditions in the workplace, exercise conditions near home, exercise place, personal laziness, physical illness, high volume physical labor requiring little exercise, busy with household chores and lack of time, busy with work and lack of energy, lack of site facilities in workplace or home, lack of exercise knowledge or guidance, lack of organization, lack of companions, economic constraints, fear of injury, weather constraints, average daily sleep time, and rest in their workplaces (Table 1). These independent variables were used for binomial logistic regression analysis.

**Table 1:** Main study factors and assignment instructions.

Factors	Assignment instructions
Whether to participate in regular exercise	1 = yes; 0 = no
Gender	1 = male; 2 = female
Age (years)	1 = under 25; 2 = 26–35; 3 = 36–45; 4 = 46–55; 5 = over 56
Education	1 = below junior college; 2 = junior college; 3 = college; 4 = undergraduate; 5 = Masters or above
Sources of joining the police	1 = police academy graduate; 2 = social recruitment; 3 = troop transfer; 4 = commercial transfer; 5 = others
Exercise conditions in the workplace	1 = very poor; 2 = poor; 3 = in general; 4 = good; 5 = very good
Exercise conditions near home	1 = very poor; 2 = poor; 3 = in general; 4 = good; 5 = very good
Places to participate in exercise	1 = exercise place in the workplace; 2 = exercise place in the neighborhood; 3 = highway street; 4 = gym; 5 = sports club; 6 = others
Personal inertia	1 = yes; 0 = no
Physical illness	
High volume physical labor requiring little exercise	
Lack of time for household chores	
Lack of energy for busy work	
Lack of exercise knowledge or guidance	
Lack of space and facilities at home or at work	
Lack of organization	
Lack of companionship	
Economic constraints	
Fear of injury	
Weather restrictions	
Average daily sleep time	1 = less than 6 h; 2 = about 6 h; 3 = about 7 h; 4 = about 8 h; 5 = more than 8 h
Rest in your workplace	1 = series of weekdays; 2 = weekend single day shifts; 3 = double days off; 4 = annual vacation breaks; 5 = unscheduled breaks

### 2.2 Procedure

A uniform questionnaire was used, and unified training was given to the surveyors before handing it out to guarantee the consistency of the survey between groups. In addition, uniformly written instructional language was used to explain the purpose and requirements of the survey to the subjects. The inclusion criteria were as follows: consent to join in the investigation, healthy, completed the questionnaire. The exclusion criteria were as follows: not consenting to join in the investigation, sick or injured, not completing the questionnaire. From April to May 2023, the questionnaire was provided online to the police officers.

### 2.3 Statistical analysis

All data were collected and exported to Microsoft Excel (2016) for further analysis. All statistical procedures were conducted using a Statistical Package for Social Sciences (SPSS Inc., versão 25.0, Chicago, IL, USA). Descriptive measures (means ± SD) were calculated for all test variables for all participants. Analysis of variance was used for comparison of the physical exercise behavior of police officers. The Chi-square test was used for comparison of police physical exercise motivation and physical exercise programs. Logistic regression analysis was used for the analysis of influencing factors; *α* = 0.05 was used as the test level.

## 3 Results

### 3.1 Physical exercise behavior

Police officers (*n* = 3,682) were recruited from 16 district public security substations in a city in eastern China. In the survey of exercise behavior, 2004 people did not participate in physical exercise regularly. There were 1,678 people who participated in physical exercise regularly, and the exercise behavior survey included three dimensions: exercise frequency, duration, and intensity. Overall, there were statistically significant differences in exercise behavior among police officers of different genders, ages, and educational levels (*p* < 0.05). The physical exercise behavior results are listed in Table 2.

**Table 2:** Comparison of physical exercise behavior of police officers.

Group	Category	People	Statistical value	Frequency of exercise per week (times)	Duration of each exercise (level)	Exercise intensity of each exercise (degree)
Gender	Male	1,484		2.23 ± 1.227	3.77 ± 0.980	2.94 ± 1.029
	Female	194		2.24 ± 1.233	3.61 ± 0.887	2.51 ± 0.829
			*F*	0.26	4.682	31.256
			*p*	>0.05	<0.05	<0.01
Age	<25	81		2.27 ± 1.235	3.93 ± 1.081	3.32 ± 1.192
	26 ~ 35	641		2.12 ± 1.202	3.80 ± 0.978	3.15 ± 0.989
	36 ~ 45	468		2.13 ± 1.168	3.75 ± 0.970	2.96 ± 0.937
	46 ~ 55	372		2.41 ± 1.263	3.73 ± 0.892	2.48 ± 0.921
	>56	116		2.59 ± 1.358	3.50 ± 1.059	2.16 ± 0.861
			*F*	6.782	2.981	49.658
			*p*	<0.01	<0.05	<0.01
Education	Below junior college	4		2.00 ± 1.414	2.25 ± 1.500	2.00 ± 2.000
	Junior college	14		2.07 ± 0.997	3.07 ± 0.917	2.00 ± 0.784
	College	395		2.40 ± 1.307	3.73 ± 0.972	2.72 ± 1.079
	Undergraduate	1,200		2.18 ± 1.205	3.78 ± 0.965	2.95 ± 0.983
	Masters or above	65		2.12 ± 1.125	3.75 ± 0.952	2.98 ± 1.023
			*F*	2.528	4.394	7.248
			*p*	<0.05	<0.01	<0.01

Data are presented as mean ± SD; *F*, *F*-value; *p*, *p*-value.

### 3.2 Physical exercise motivation

Among the 1,678 police officers who chose to exercise regularly, the survey found that their motivations for physical exercise were as follows: 86.71% to keep fit; 50.54% for stress relief; 15.26% for work needs; 9.71% for bodybuilding and muscle building; 28.67% for weight loss and shaping; 5.18% for skill enhancement; 2.56% for social expansion; and 3.87% for other reasons. Among them, the main motives for men were body building (*n* = 1,288, 88.5%), stress relief (*n* = 741, 87.4%), and weight loss and shaping (*n* = 387, 80.5%). The primary motivations of women were body building (*n* = 145, 11.5%), stress relief (*n* = 107, 12.6%), and weight loss and shaping (*n* = 94, 19.5%). There was a significant difference (*p* < 0.05) in (i) the motivation of work, weight loss and shaping, and expanding social interaction among police officers of different genders; (ii) in the exercise motivation of police officers of diversified ages; and (iii) in exercise motivation for stress relief, bodybuilding and muscle building, and other motivations for police officers of different educational levels (Table 3).

**Table 3:** Comparison of police physical exercise motivation.

Group	Category	People	Statistical value	Keeping fit	Stress relief	Work needs	Fitness and muscle building	Weight loss and shaping	Enhancement of skills	Expanding social life	Other motivations
Gender	Male	1,484		1,288 (88.5)	741 (87.4)	248 (96.9)	144 (88.3)	387 (80.5)	83 (95.4)	43 (100)	60 (92.3)
	Female	194		145 (11.5)	107 (12.6)	8 (3.1)	19 (11.7)	94 (19.5)	4 (4.6)	0 (0)	5 (7.7)
			χ^2^	0.953	2.679	21.077	0.883	41.228	5.061	6.422	1.832
			*p*	>0.05	>0.05	<0.01	>0.05	<0.01	>0.05	<0.05	>0.05
Age	<25	81		67 (6.3)	40 (4.7)	17 (6.6)	18 (11.0)	27 (5.6)	6 (6.9)	2 (4.7)	2 (3.1)
	26 ~ 35	641		548 (37.7)	323 (38.1)	119 (46.5)	97 (59.5)	221 (45.9)	44 (50.6)	18 (41.9)	15 (23.1)
	36 ~ 45	468		408 (28.0)	246 (29.0)	59 (23.0)	37 (22.7)	153 (31.8)	23 (26.4)	14 (32.6)	16 (24.6)
	46 ~ 55	372		328 (22.5)	190 (22.4)	44 (17.2)	10 (6.1)	67 (13.9)	11 (12.6)	5 (11.6)	23 (35.4)
	>56	116		104 (7.1)	49 (5.8)	17 (6.6)	1 (0.6)	13 (2.7)	3 (3.4)	4 (9.3)	9 (13.8)
			χ^2^	31.974	31.766	42.252	102.380	80.179	38.252	30.985	42.726
			*p*	<0.01	<0.01	<0.01	<0.01	<0.01	<0.01	<0.01	<0.01

Data are presented as n (%); χ^2^, Chi-Square; *p*, *p*-value.

### 3.3 Police physical exercise programs

Among the 1,678 police officers who chose to exercise regularly, the survey found that their choices of physical exercise types included the following: running (*n* = 944, 56.26%), walking (*n* = 697, 41.54%), ball games (*n* = 421, 25.09%), equipment (*n* = 391, 23.3%), cycling (*n* = 181, 10.79%), rope skipping (*n* = 178, 10.61%), yoga (*n* = 54, 3.22%), tai chi chuan (*n* = 20, 1.19%), square dance (*n* = 15, 0.89%), and others (*n* = 342, 20.38%). Except for the cycling and tai chi programs, there was a significant difference (*p* < 0.05) in the choice of exercise programs by gender and age (Table 4).

**Table 4:** Comparison of police physical exercise programs.

Group	Category	People	Statistical value	Walking	Running	Cycling	Balls	Equipment Fitness	Square dance	Tai chi chuan	Yoga	Rope jumping	Others
Gender	Male	1,484		596 (85.5)	875 (92.7)	165 (91.2)	402 (95.5)	370 (94.6)	8 (53.3)	18 (90.0)	17 (31.5)	155 (87.1)	282 (82.5)
	Female	194		101 (14.5)	69 (7.3)	16 (8.8)	19 (4.5)	21 (5.4)	7 (46.7)	2 (10.0)	37 (68.5)	23 (12.9)	60 (17.5)
			χ^2^	10.491	37.533	2.293	27.107	19.233	18.402	0.928	170.909	1.227	15.322
			*p*	<0.01	<0.01	>0.05	<0.01	<0.01	<0.01	>0.05	<0.01	>0.05	<0.01
Age	<25	81		20 (2.9)	59 (6.3)	8 (4.4)	21 (5.0)	30 (7.7)	2 (13.3)	1 (5.0)	4 (7.4)	15 (8.4)	17 (5.0)
	26 ~ 35	641		151 (21.7)	429 (45.4)	79 (43.6)	205 (48.7)	201 (51.4)	3 (20.0)	4 (20.0)	20 (37.0)	93 (52.2)	139 (40.6)
	36 ~ 45	468		191 (27.4)	291 (30.8)	53 (29.3)	122 (29.0)	98 (25.1)	0 (0)	4 (20.0)	16 (29.6)	48 (27.0)	97 (28.4)
	46 ~ 55	372		238 (34.1)	145 (15.4)	31 (17.1)	61 (14.5)	53 (13.6)	5 (33.3)	9 (45.0)	13 (24.1)	21 (11.8)	69 (20.2)
	>56	116		97 (13.9)	20 (2.1)	10 (5.5)	12 (2.9)	9 (2.3)	5 (33.3)	2 (10.0)	1 (1.9)	1 (0.6)	20 (5.8)
			χ^2^	286.256	189.011	32.703	72.650	97.123	51.373	35.207	31.129	66.590	30.173
			*p*	<0.01	<0.01	<0.01	<0.01	<0.01	<0.01	<0.01	<0.01	<0.01	<0.01

Data are presented as n (%); χ^2^, Chi-Square; *p*, *p*-value.

### 3.4 Logistic regression analysis of factors influencing physical exercise behavior

The results showed that age, source of entry into the police, exercise conditions in the workplace, exercise conditions near the home, participation in exercise places, high volume physical labor, lack of exercise knowledge or guidance, lack of organization, lack of companions, economic condition restrictions, weather restrictions, and rest situation factors in the workplace were all related to police physical exercise behavior (*p* < 0.05; Table 5).

**Table 5:** Univariate logistic regression analysis of factors influencing physical exercise behavior (*n* = 3,682).

Independent variables	*β*	Standard error	Wald χ^2^	*p*	OR (95% CI)
Gender	0.132	0.113	1.371	0.242	1.142 (0.915–1.425)
Age	0.170	0.043	15.754	<0.001	1.185 (1.090–1.288)
Education	0.060	0.076	0.633	0.426	1.062 (0.915–1.233)
Source of joining the police	−0.117	0.036	10.382	0.001	0.890 (0.829–0.955)
Exercise conditions in workplace	−0.190	0.039	23.941	<0.001	0.827 (0.766–0.892)
Exercise conditions near home	−0.405	0.048	70.447	<0.001	0.667 (0.607–0.733)
Exercise place	0.135	0.022	37.901	<0.001	1.144 (1.096 ~ 1.194)
Personal inertia	−0.071	0.130	0.297	0.586	0.932 (0.722–1.202)
Physical illness	−0.036	0.143	0.062	0.803	0.965 (0.730–1.277)
High volume physical labor requiring little exercise	−0.425	0.178	5.694	0.017	0.654 (0.462–0.927)
Lack of time for household chores	0.137	0.134	1.049	0.306	1.147 (0.882–1.490)
Lack of energy for busy work	0.071	0.137	0.268	0.605	1.074 (0.820–1.405)
Lack of exercise knowledge or guidance	−0.561	0.137	16.847	<0.001	0.570 (0.436–0.746)
Lack of space and facilities at home or at work	−0.189	0.144	1.727	0.189	0.828 (0.624–1.097)
Lack of organization	−0.363	0.146	6.228	0.013	0.695 (0.523–0.925)
Lack of companionship	0.505	0.146	11.994	0.001	1.657 (1.245–2.205)
Economic constraints	−0.424	0.169	6.333	0.012	0.654 (0.470–0.910)
Fear of injury	0.030	0.158	0.037	0.848	1.031 (0.756–1.405)
Weather restrictions	−1.127	0.150	56.586	<0.001	0.324 (0.241–0.434)
Average daily sleep time	−0.080	0.043	3.580	0.058	0.923 (0.849–1.003)
Rest in your workplace	0.057	0.026	4.753	0.029	1.059 (1.006–1.115)

*β, beta-coefficient; Wald χ2, Wald chi-square; p, p-value; OR, odds ratio; CI, confidence interval.*

Factors whose variables were statistically significant in the results of univariate logistic regression analysis were subjected to multivariate logistic regression analysis. The results showed that the source of joining the police, exercise conditions in the workplace, exercise conditions near the home, high volume physical labor, lack of organization, economic conditions restrictions, and weather restrictions factors were all relevant to police physical exercise behavior (*p* < 0.05 and odds ratio > 1; Table 6).

**Table 6:** Multivariate logistic regression analysis of factors influencing physical exercise behavior (*n* = 3,682).

Independent variables	*β*	Standard error	Wald χ^2^	*p*	OR (95% CI)
Age	−0.163	0.038	18.893	<0.001	0.849 (0.789–0.914)
Source of joining the police	0.383	0.047	66.254	<0.001	1.467 (1.338–1.609)
Exercise conditions in workplace	0.067	0.009	59.604	<0.001	1.069 (1.051–1.088)
Exercise conditions near home	0.116	0.036	10.588	0.001	1.123 (1.047–1.205)
Exercise place	−0.139	0.021	41.835	<0.001	0.870 (0.835–0.908)
High volume physical labor requiring little exercise	0.316	0.142	4.936	0.026	1.372 (1.038–1.813)
Lack of exercise knowledge or guidance	0.160	0.100	2.555	0.110	1.173 (0.964–1.428)
Lack of organization	0.347	0.100	12.111	0.001	1.415 (1.164–1.720)
Lack of companionship	−0.539	0.101	28.502	<0.001	0.583 (0.479–0.711)
Economic constraints	0.364	0.130	7.783	0.005	1.439 (1.114–1.858)
Weather restrictions	1.075	0.098	120.783	<0.001	2.929 (2.418–3.548)
Rest situation in your workplace	−0.061	0.026	5.566	0.018	0.941 (0.895–0.990)

*β, beta-coefficient; Wald χ2, Wald chi-square; p, p-value; OR, odds ratio; CI, confidence interval.*

## 4 Discussion

The aim of this study was to investigate and analyze the physical exercise behavior characteristics, health cognition, and influencing factors of police officers, to provide insights about the physical health of police officers and the optimal development of police careers.

First, this study on the physical exercise behavior of police officers revealed low exercise participation. More than half (54.43%) of the police officers indicated that they did not participate in physical exercise regularly. This shows that the level of awareness and subjective willingness of police officers to promote health through physical exercise needs to be strengthened. It also demonstrates that police officers objectively had a low level of participation in exercise because of various barriers to physical exercise. This finding is similar to that of studies in other Chinese provinces (24, 25).

Second, the specific exercise behavior was of long time duration, but with low frequency and intensity. Specifically, 64.78% of the police officers exercised less than three times a week and only 66.81% of police officers exercised for more than 30 min each time. Furthermore, 79.68% of police officers exercised at low to medium intensity. Moreover, exercise behavior decreased with age. These findings are similar to those of a study carried out on police in Finland (18). This may be related to the fact that the attainable standard in the physical attainment test of the public security system declines with age, leading to a decrease in the driving force of police exercise. In situations where police officers are required to carry out vigorous physical activity, such as pursuing criminal suspects, it may cause damage to their physical health and physiological function due to the low level of normal physical exercise. Officers may also have to respond quickly to situations with a dramatic increase in physical demands, moving from passive energy expenditure (80–90% of officers) (1.6 MET) to vigorous energy expenditure (10–20% of officers) police officers (12.5 MET).

Third, exercise programs were diverse but relatively concentrated. The exercise of police officers in the current study was mainly running, whereas a study by Sörensen (18) found that walking was the most common form of exercise for police officers. The possible reason might be that running is not restricted by sports facilities, economic conditions, or venue companions, and is, therefore, easier for police officers to achieve after a busy day.

As to health cognition, keeping fit and stress relief were the main motivations. A tentative explanation is that the mean level of police occupational physical activity is very low (17). However, police officers need to maintain a strong and healthy body in order to face decades of a police career, to avoid strain because of physical overload, and to be able to cope with various personal injuries. These strains and injuries might be brought about by police enforcement combat confrontations such as assault and making arrests. Studies of the stressors related to public security work, such as work tasks, career achievements, interpersonal relationships, and other aspects (26) have found that this type of work can cause psychological stress, which can lead to depression and anxiety (27). Exercise can relieve both depressive and anxiety disorders (28, 29). The results indicate that the officers recognized that physical exercise could affect their ability to cope with stressful situations, which is consistent with the research of Kuhns (30).

Logistic regression analysis indicated that the factors affecting the physical exercise behavior of police officers mainly included the source of joining the police, exercise conditions in the workplace, exercise conditions near home, high volume physical labor, lack of organization, lack of companions, economic condition restrictions, weather restrictions, and the rest situation in the workplace. Among these factors, exercise conditions, lack of organization, and economic condition restrictions were consistent with the findings of Feng Caixia’s study on members of the general public (31) and Soroka’s study on police officers and cadets (13). However, the findings were partially different from those of L Sörensen who reported that a lack of knowledge was a factor (18).

What should not be neglected is that police officers are not only civil servants but also front-line combatants who arrest criminals and protect the lives and property of citizens; therefore, more attention should be paid to police officers’ physical fitness. However, the regression results show that a significant proportion of police officers still have the misconception that physical labor replaces physical exercise. In fact, Pescatello point out that physical labor is just general physical activity not active physical exercise (32). Weather restrictions were one of the factors affecting public security physical exercise in eastern cities of China, probably because of the humid and rainy climate. Running, which was the first choice in the survey of police physical exercise items, is affected by bad weather such as rain and haze. Different sources of joining the police had a large impact on physical exercise, as shown by the fact that police officers who joined the police after graduating from the police academy performed better in terms of physical exercise behavior and health cognition, and had a stronger awareness of physical exercise. This might be related to their study and training life in the police academy. In the current police academies in China, police physical education courses are mandatory professional foundation courses and physical fitness is viewed as a practical professional ability to be cultivated.

Lack of energy after busy work accounted for the largest proportion (75.29%) as a factor influencing whether to participate in physical exercise regularly. The impact of this factor is commonly encountered by police groups and has also been discussed in several studies. However, the regression data revealed that this factor did not show statistical significance, in other words, it was not the main influencing factor on whether police officers regularly participated in physical exercise. This finding is inconsistent with that of previous studies in China but is consistent with Soroka’s work in Poland (13). The reason is that both regular and infrequent exercisers considered “lack of energy due to busy work” as a factor affecting the regularity of exercise participation, but in fact it did not become the main factor affecting their participation in physical exercise. In other words, although police officers who exercised regularly, like those who exercised infrequently, believed that being busy at work largely affected physical exercise, they still maintained a high level of motivation and participation. Through face-to-face communication during the questionnaire distribution process, many police officers also expressed the fact that there was a problem of prioritizing the importance of doing different tasks. The degree of participation in physical exercise depended on the importance of the exercise itself, and those who liked to exercise would find time to exercise even if they were busy. By contrast, those who did not like to exercise could always find excuses to avoid it.

### 4.1 Limitations

This study was a cross-sectional study, and we did not consider the effect of confounding variables. In future studies, we plan to expand our sample by representing different provinces and areas.

## 5 Conclusion

This study of the physical exercise behaviors of police officers revealed characteristics of low participation, various exercise items, and exercise motivation focused on keeping fit and relieving stress. Police officers’ physical exercise behavior was influenced by age, source of joining the police, exercise conditions in their workplaces, exercise conditions near their homes, places to participate in exercise, high volume physical labor during work resulted in little time for exercise, lack of organization, lack of companions, economic condition restrictions, weather restrictions, and rest conditions in their workplaces.

Therefore, in view of the above factors, fitness venues or gyms should be built appropriately in each workplace so that police officers can complete small, daily, fragmented, and diversified physical exercise activities during their breaktimes. In addition, internal public security sports events should be held to increase the participation of police officers in physical exercise. These events should advocate participation and competition for all staff to create a positive and harmonious police camp atmosphere. At the same time, considering the high fees of urban sports venues, preferential measures should be taken to reduce or provide free memberships of commercial sports venues to police officers and their families.

## Data Availability

The raw data supporting the conclusions of this article will be made available by the authors, without undue reservation.
